# Hemodynamic change in patients with hypertrophic obstructive cardiomyopathy before and after alcohol septal ablation using 4D flow magnetic resonance imaging: a retrospective observational study

**DOI:** 10.1186/s12872-021-02003-8

**Published:** 2021-04-20

**Authors:** Kenichiro Suwa, Keitaro Akita, Keisuke Iguchi, Takasuke Ushio, Yuichiro Maekawa

**Affiliations:** 1 Division of Cardiology, Internal Medicine 3, Hamamatsu University School of Medicine, 1-20-1 Handayama, Higashi-ku, 431-3192 Hamamatsu, Japan; 2 Department of Radiology, Hamamatsu University School of Medicine, 1-20-1 Handayama, Higashi-ku, 431-3192 Hamamatsu, Japan

**Keywords:** 4D flow MRI, Alcohol septal ablation, Hemodynamics, Hypertrophic obstructive cardiomyopathy

## Abstract

**Background:**

The hemodynamics in the left ventricle (LV) and the ascending aorta (AAO) before and after alcohol septal ablation (ASA) in patients with hypertrophic obstructive cardiomyopathy (HOCM) is elucidated. Our objective was to evaluate the pattern changes in AAO and intra-LV flow assessed by four-dimensional (4D) flow magnetic resonance imaging (MRI) before and after ASA and to clarify the association between 4D flow MRI-derived hemodynamic characteristics and the peak pressure gradient (PPG) in patients with drug-refractory HOCM.

**Methods:**

In this retrospective observational study, 11 patients with HOCM underwent 4D flow MRI before and a week after ASA. The 4D flow MRI included blood flow visualization and quantification using streamline images. The combined score of vortex and helix in AAO was analyzed. The duration and phase count of the AAO vortex or helix flow and the size of the intra-LV anterior vortex were quantified. The correlation between the changes in hemodynamics and the resting PPG at LV outflow tract was also analyzed. We used the paired t-test for the comparison between before and after ASA and the Pearson’s correlation coefficient for the analysis.

**Results:**

The combined score for the incidence of vortex and/or helix flow in AAO after ASA was significantly lower than that before ASA (1.45 ± 0.52 vs. 1.09 ± 0.30, *p* = 0.046). The duration (744 ± 291 ms vs. 467 ± 258 ms, *p* < 0.001) and phase count (14.8 ± 4.4 phases vs. 10.5 ± 5.8 phases, *p* < 0.001) of the vortex or helix flow in AAO were significantly decreased after ASA. The LV anterior vortex area after ASA was significantly larger than that before ASA (1628 ± 420 mm^2^ vs. 2974 ± 539 mm^2^, *p* = 0.009). The delta phase count of the AAO vortex or helix before and a week after ASA was significantly correlated with delta PPG before and a week after ASA (R = 0.79, *p* = 0.004) and with delta PPG before and 6 months after ASA (R = 0.83, *p* = 0.002).

**Conclusions:**

Lower vortex or helix flow in AAO and larger diastolic vortex flow in LV were observed after ASA, which suggests the possibility to detect the changes of aberrant hemodynamics in HOCM.

**Supplementary Information:**

The online version contains supplementary material available at 10.1186/s12872-021-02003-8.

## Background

Left ventricular outflow tract (LVOT) obstruction (LVOTO) is one of the important determinants of major adverse cardiac events in hypertrophic cardiomyopathy (HCM) [1–3]. The relief of obstruction improves the short- or long-term prognosis of HCM [4, 5]. Alcohol septal ablation (ASA) is a method of reducing LVOTO and alleviating heart failure symptoms in patients with hypertrophic obstructive cardiomyopathy (HOCM) [6–8]. There is some evidence that ascending aorta (AAO) flow is disease-specific in valvular heart disease and cardiomyopathy [9–12]. Four-dimensional (4D) flow magnetic resonance imaging (MRI) can demonstrate that flow derangement in AAO can be frequently observed in patients with HOCM compared to healthy volunteers and may also be associated with the severity of LVOTO [13]. 4D flow MRI can also achieve flow visualization and quantification in AAO, and evaluate aortic hemodynamics in high spatial and temporal resolutions [14]. Therefore, in the present study, we aimed to evaluate the pattern changes in AAO and intra-left ventricular flow before and after ASA and to elucidate the association between 4D flow MRI-derived hemodynamic characteristics and the LVOT peak pressure gradient (PPG) in patients with drug-refractory HOCM.

## Methods

### Patients

**Fig. 1:**
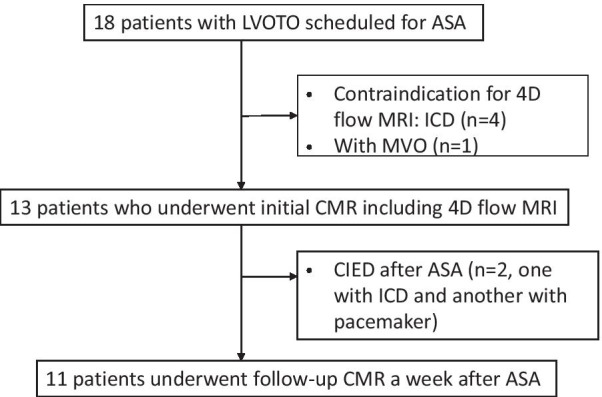
Patient selection. Of the 18 HOCM patients with LVOTO who were scheduled to undergo ASA, four patients with contraindications for 4D flow MRI due to ICD, and one patient with MVO were excluded. The remaining 13 patients underwent an initial 4D flow MRI and proceeded to the ASA. After the exclusion of two patients who required CIED implantation after ASA, the remaining 11 patients who underwent follow-up CMR study a week after ASA were recruited for the analysis. Abbreviations: ASA, alcohol septal ablation; CIED, cardiac implantable electrical device; CMR, cardiac magnetic resonance; HOCM, hypertrophic obstructive cardiomyopathy; ICD, implantable cardioverter defibrillator; LVOTO: left ventricular outflow tract obstruction; MRI, magnetic resonance imaging; MVO, mid-ventricular obstruction

This retrospective observational study included 18 patients diagnosed with HOCM by transthoracic echocardiography (TTE) in our institute from February 1, 2018 to May 31, 2020. The patient selection is presented in Fig. 1. All patients underwent ASA for drug-refractory HOCM. HOCM is defined as a maximum left ventricle (LV) thickness ≥ 15 mm or ≥ 13 mm with a family history of HCM and LV cavity obliteration with a pressure gradient > 30 mmHg at rest or under provocation by TTE [15]. Four patients who had cardiac implantable electrical devices (CIED) compatible only with a 1.5T MRI scanner were excluded from the analysis because a 3T scanner was required for 4D flow MRI in our institute. Patients with concomitant mid-ventricular obstruction were also excluded. As a result, the initial 4D flow MRI analysis was performed in 13 patients with HOCM scheduled for ASA. Follow-up cardiac MRI a week after ASA was cancelled for two patients who required pacemaker or implantable cardioverter defibrillator implantation because of sustained complete atrioventricular block and/or ventricular tachycardia, which are contraindications for MRI examination. Finally, 11 patients who underwent cardiac MRI before and a week after ASA were included in the study. This study complied with the principles of Declaration of Helsinki and was approved by the Ethics Committee of Hamamatsu University School of Medicine. All patients provided written informed consent.

### Transthoracic echocardiography

All patients underwent TTE for the initial evaluation of HOCM and the assessment of the intervention in a week and six months (6 M) after ASA. TTE parameters included LV end-diastolic dimension (LVEDD), LV end-systolic dimension (LVESD), maximum interventricular septal thickness (Max-IVST), left atrial dimension (LAD), left atrial volume index (LAVI), resting PPG at LVOT, the incidence of moderate or severe mitral regurgitation (MR), and the length of the anterior and posterior leaflets of the mitral valve. The systolic anterior motion (SAM) of the mitral valve leaflet was evaluated according to the following grading: G0, without SAM; G1, no mitral leaflet-septal contact with a systolic minimum distance > 10 mm; G2, no mitral leaflet-septal contact with a systolic minimum distance < 10 mm; G3, brief mitral leaflet-septal contact; and G4, prolonged mitral leaflet-septal contact [16]. The anterior and posterior leaflets of the mitral valve were also measured during TTE before ASA.

### Cardiac MRI

Cardiac MRI (Discovery MR750 or MR750w, GE Healthcare, Waukesha, USA) was performed for all patients before and a week after ASA with a maximum gradient strength of 50 mT/m (MR750) or 44 mT/m (MR750w) and a maximum slew rate of 200 mT/m/ms (both MR750 and MR750w). A commercially available 32-channel phased array body coil for MR750, or the geometry embedding method phased array coil for MR750w were applied. For the clinical purposes of assessment of chamber size and extent of scar, two-dimensional (2D) fast imaging employing steady-state acquisition (FIESTA) based on a steady-state free precession sequence for cine images and inversion recovery prepared fast gradient echo sequence for late gadolinium enhancement (LGE) images were acquired in the short, vertical long-axis and horizontal long-axis orientations with a slice thickness/gap of 10 mm/0 mm. A bolus injection of 0.1 mmol/kg gadobutrol (Gadovist, Bayer AG, Berlin, Germany) using a power injector (Sonic Shot GX, Nemoto Kyorindo Co., Tokyo, Japan) was administered at an injection rate of 2.0 mL/s followed by 20 mL of saline at the same injection rate.

### Conventional cardiac MRI

Conventional cardiac MRI images were analyzed using Cardiac VX (GE Healthcare, Waukesha, USA). Max-IVST in diastole (Max-IVSTd) and systole (Max-IVSTs), LV end-diastolic and LV end-systolic volume indices (LVEDVI and LVESVI), LV stroke volume index (LVSVI), LV ejection fraction (LVEF), LV mass index (LVMI), right ventricular (RV) end-diastolic and RV end-systolic volume index (RVEDVI and RVESVI), and RV ejection fraction (RVEF) were quantified using short-axis images of 2D FIESTA. Papillary muscles and trabeculations were included in the blood pool. LGE mass and a fraction of LGE to LV mass (%LGE) were analyzed by measuring regions with signal intensity > 6 standard deviations (SD) above the nulled remote myocardium. Whole lesions with microvascular obstruction were manually included in the LGE mass.

### 4D flow MRI acquisitions

Retrospective electrocardiography-gated 4D flow MRI data were acquired using a respiratory navigator with full volumetric coverage of the whole heart and ascending thoracic aorta. The imaging parameters for 4D flow MRI were as follows: repetition time (TR): 4.7 ms, echo time (TE): 2.4 ms, flip angle (FA): 15°, number of excitations (NEX): 4, field of view (FOV): 32–36 cm, matrix of 224 ⋅ 224, thickness: 2 mm, partitions: 60, phases during one cardiac cycle: 20, velocity sensitivity (VENC): 550 cm/s, and receiver bandwidth (RBW): 62.5 kH. The data were reconstructed using autocalibrating reconstruction for Cartesian sampling with a reduction factor of 2 on a personal computer (Intel Xeon E3-1270 [3.4 GHz/Quad-core] DDr3, 16GB ECC, Linux). For geometric information for 4D flow MRI, multiphase contrast-enhanced 3D fast spoiled gradient recalled acquisition in the steady state (FSPGR) MR angiography (MRA) was performed just after injection of contrast medium and before LGE scan. Imaging parameters for coronal MRA were as follows: TR: 3.2 ms, TE: 1 ms, FA: 12°, NEX: 1, FOV: 32–36 cm, size of the reconstructed matrix with the aid of zero-fill interpolation: 512, RBW: 83.3 kHz, and imaging time: 33 s for four phases.

### 4D flow MRI postprocessing

4D flow MRI and 3D FSPGR MRA datasets were formatted by digital imaging and communications in medicine for postprocessing. The 3D segmentation of heart and vessel structure and visualization and quantification of hemodynamic measurements for 4D flow MRI were performed using Flova (R’-Tech, Hamamatsu, Japan). The regions of interest, including the LA, LV, and thoracic aorta, were determined at the R wave peak using the region-growing method. Their shapes were rendered by the marching-cubes method. The 3D flow information was interpolated with a spatial resolution of 2 × 2 × 2 mm using 3D datasets. Three-D streamline images with a cardiac cycle divided into 20 phases were created using the Runge–Kutta method to visualize and quantify hemodynamics.

### Hemodynamic assessment by 4D flow MRI

**Fig. 2:**
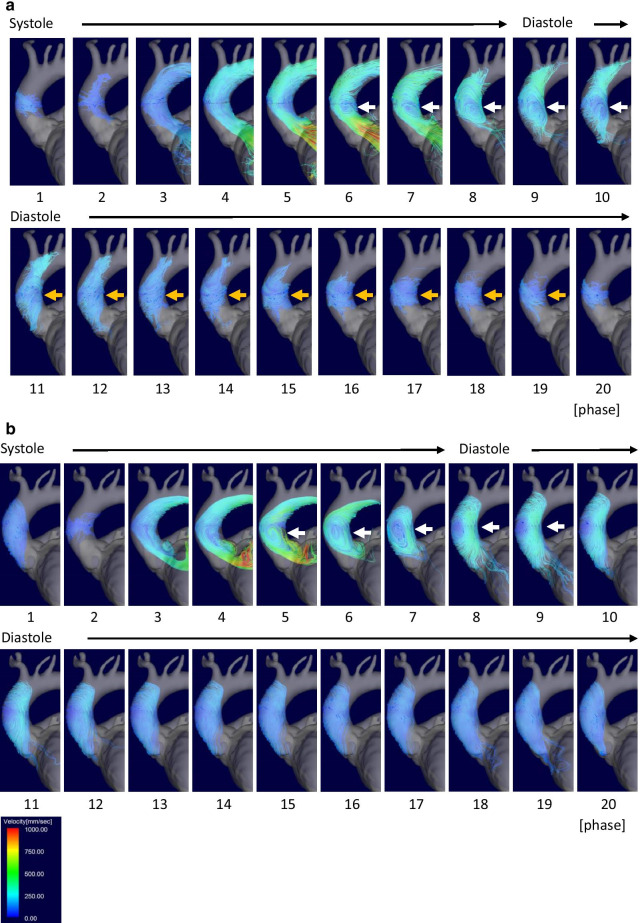
Serial streamline images in ascending aorta before and a week after ASA. The vortex flow (white arrows) appeared in the mid-ascending aorta in the late systole to early diastole both before (**a**) and a week after ASA (**b**). In images before ASA, the vortex flow changed to helix flow (yellow arrows) at mid diastole and continued to late diastole (**a**). In contrast, the vortex or helix flow disappeared in mid to late diastole after ASA (**b**). The cardiac phases started from early systole (phase 1) and ended at late diastole (phase 20). Abbreviation: ASA, alcohol septal ablation

**Fig. 3:**
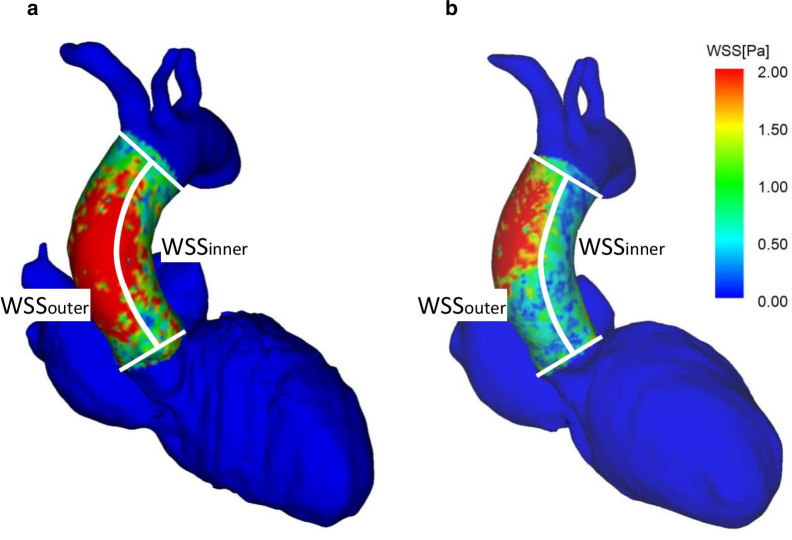
Three-dimensional wall shear stress images before and a week after ASA. The peak systolic WSS was visualized before (**a**) and a week after ASA (**b**). Area-averaged peak systolic WSS was calculated in WSS_outer_ and WSS_inner_ curvatures of the ascending aorta (see the dividing white line). Abbreviations: ASA, alcohol septal ablation; WSS, wall shear stress; WSS_inner_, wall shear stress at inner curvature of aortic arch; WSS_outer_, wall shear stress at outer curvature of aortic arch

**Fig. 4:**
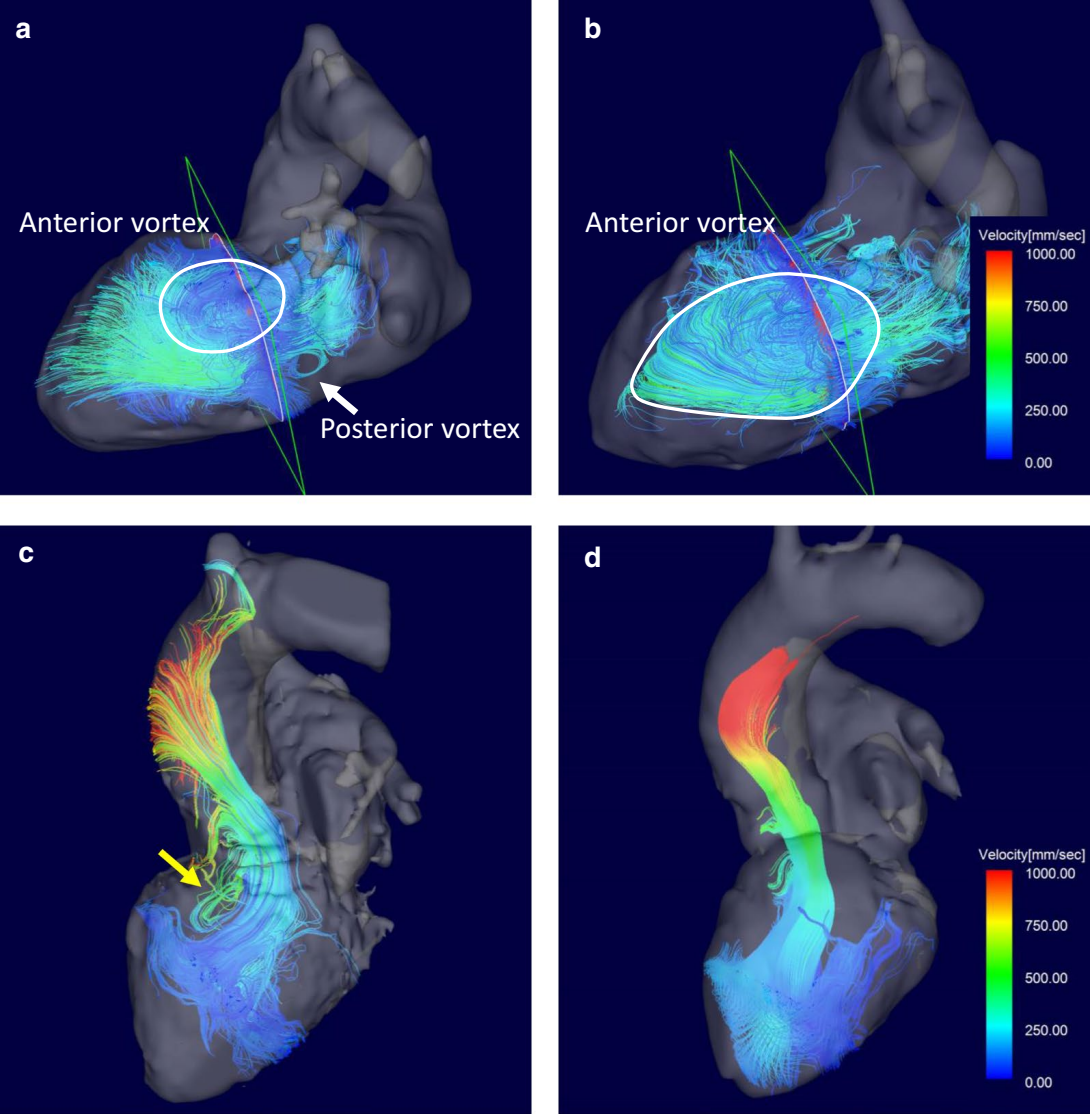
Streamline images of diastolic LV vortex before and a week after ASA. Diastolic LV vortex flow was observed in the left lateral view of the heart. Typically, the anterior vortex (white circle) appeared at the basal LV before ASA (**a**), which grew and became larger after ASA (**b**). The posterior vortex (white arrow) was visualized just below the LV inflow at the basal LV (**a**). In contrast, systolic LV vortex flow was observed in the anterior view of the heart. The systolic vortex just above LVOT (yellow arrow) was typically observed before ASA (**c**) and disappeared after ASA (**d**). Abbreviations: ASA, alcohol septal ablation; LV, left ventricle; LVOT, left ventricular outflow tract

Aorta flow patterns were evaluated by the presence of vortex or helix flow, i.e., point 1 for the presence and point 0 for absence. Vortex flow was defined as revolving particles around an axis orthogonal to the vessel centerline. Helix flow was defined as rotational motion around the longitudinal axis of the vessel centerline. The combined measurement of vortex and helix existence (Vortex + Helix) was also calculated as the sum of the points. We also measured the duration and phase count of the vortex and/or helix flow within a cardiac cycle (Fig. 2). In patients with a vortex in the aorta, the area of the vortex was measured. As shown in Fig. 3, the 3D wall shear stress (WSS) at peak systole in the AAO from the ST junction to the origin of the brachiocephalic artery was quantified for the outer (WSS_outer_) and inner curvature of the aortic arch (WSS_inner_). For the intra-LV hemodynamics, diastolic LV inflow was visualized, and the presence of a concomitant vortex ring was investigated. LV inflow was observed from the left lateral side of the heart. Both the anterior vortex generated above the mitral valve rotated clockwise (Fig. 4a and b, white circle) and the posterior vortex generated below the mitral valve rotated in a counterclockwise direction (Fig. 4a, white arrow) were evaluated to determine whether they were present or not. The maximum area of the diastolic LV vortex observed from the left lateral side was also quantified. We evaluated systolic LV outflow and the presence of vortex flow just above LVOT (Fig. 4c, yellow arrow). LV outflow was observed from the anterior side of the heart (Fig. 4c and d). To assess the association between hemodynamic parameters and severity of HOCM, the differences in 4D flow MRI-derived hemodynamic parameters before and a week after ASA were calculated and compared with the difference in LVOT PPG. The 4D flow MRI analysis was repeated in all patients by a second observer (K.A.) independently for the assessment of interobserver variabilities. The determination of the presence of vortex and helix in AAO and the vortex size in AAO and LV were performed independently.

### Statistical analysis

Continuous data are expressed as means ± SD for normal distribution or as medians with interquartile ranges for non-normal distribution. Categorical data are shown as numbers and percentages. For the comparison between before and after ASA, the paired t-test for normal distribution and the Wilcoxon signed-rank test for non-normal distribution were used. Categorical data were compared using Fisher’s exact test. The Pearson’s correlation coefficient was calculated for correlation analysis. The interobserver variabilities for the determination of the presence of vortex and helix in AAO, systolic vortex above LVOT, the vortex size in AAO, and diastolic LV vortex size were evaluated by calculating Cohen’s kappa coefficient or intraclass correlation coefficients (ICC). All statistical analyses were performed using SPSS statistics, version 25 (IBM Corporation, Armonk, NY, USA).

## Results

### Baseline characteristics and details of the procedure characteristics

The baseline characteristics of the participants are listed in Table 1. The raw data are shown in Additional file 1: Table S1. The mean age was 67 ± 12 years, and 72.7% of all patients were women. The prevalence of a family history of HCM and paroxysmal atrial fibrillation was 27.3% and 18.2%, respectively. Details of the procedure characteristics are listed in Table 2. Resting LVOT PPG decreased 6 M after ASA (108.7 ± 68.8 mmHg before ASA vs. 28.0 ± 19.0 mmHg 6 M after ASA, *p* = 0.014). No deaths or strokes were observed, although two patients had a transient complete atrioventricular block complication during hospitalization. New York Heart Association (NYHA) class III or IV heart failure 6 M after ASA (7 [63.6%] before ASA vs.1 [9.1%] after ASA, *p* = 0.024) and the serum N-terminal prohormone of B-type natriuretic peptide (NT-proBNP) level 6 M after ASA (1610.5 [211.0–5060.0] pg/mL before ASA vs. 315.0 [266.0–1035.0] pg/mL after ASA, *p* = 0.003) were significantly improved compared to those before ASA. Concerning TTE variables, Max-IVST by TTE was significantly reduced 6 M after ASA (17.6 ± 4.4 mm vs. 16.2 ± 3.9 mm, *p *= 0.008). SAM grades 6 M after ASA were significantly mitigated compared to those before ASA (G0 [9.1%], G1 [0.0%], G2 [9.1%], G3 [45.4%], and G4 [36.3%] before ASA vs. G0 [9.1%], G1 [18.2%], G2 [63.6%], G3 [0.0%], and G4 [9.1%] after ASA, *p* = 0.006), while the other parameters demonstrated no change.

**Table 1:** Baseline characteristics

Number of patients	n = 11
Age, years	67.2 ± 12.2
Male, n (%)	3 (27.3)
BMI, kg/m^2^	22.4 ± 2.5
NYHA class III or IV, n (%)	7 (63.6)
Syncope, n (%)	1 (9.1)
Hypertension, n (%)	4 (36.4)
Diabetes mellitus, n (%)	0 (0.0)
Dyslipidemia, n (%)	4 (36.4)
COPD, n (%)	0 (0.0)
Current smoking, n (%)	0 (0.0)
CPA, n (%)	0 (0.0)
Stroke, n (%)	0 (0.0)
Atrial fibrillation, n (%)	2 (18.2)
CIED implantation, n (%)	0 (0.0)
Family history of SCD, n (%)	1 (9.1)
Family history of HCM, n (%)	3 (27.3)
Medication	
Na channel blockers, n (%)	8 (72.7)
Beta blockers, n (%)	10 (90.9)
Calcium channel antagonists, n (%)	7 (63.6)
ACE-Is/ARBs, n (%)	1 (9.1)
Amiodarones, n (%)	1 (9.1)
OACs, n (%)	2 (18.2)
Laboratory data	
NT-proBNP, pg/mL	1610.5 (211.0–5060.0)
Echocardiographic variables	
LVEDD, mm	41.4 ± 4.0
LVESD, mm	24.6 ± 3.8
Max-IVST, mm	17.6 ± 4.4
LAD, mm	40.5 ± 5.1
LAVI, mL/m^2^	58.8 ± 16.9
Resting LVOT PPG, mmHg	108.7 ± 68.8
Moderate or severe MR, n (%)	7 (63.6)
Anterior leaflet length of MV, mm	25.9 ± 4.7
Posterior leaflet length of MV, mm	17.4 ± 4.0
SAM grade of MV	
G0, n (%)	1 (9.1)
G1, n (%)	0 (0.0)
G2, n (%)	1 (9.1)
G3, n (%)	5 (45.4)
G4, n (%)	4 (36.3)

Data are expressed as mean ± standard deviation or median (interquartile range) and number (%)

ACE-Is, angiotensin-converting enzyme inhibitors; ARBs, angiotensin receptor blockers; BMI, body mass index; CIED, cardiac implantable electrical device; COPD, chronic obstructive pulmonary disease; CPA, cardiopulmonary arrest; G0–4, grade 0–4; HCM, hypertrophic cardiomyopathy; LAD, left atrial dimension; LAVI, left atrial volume index; LVEDD, left ventricular end-diastolic dimension; LVESD, left ventricular end-systolic dimension; LVOT PPG, peak pressure gradient at left ventricular outflow tract; Max-IVST, maximum interventricular septal thickness; MR, mitral regurgitation; MV, mitral valve; NT-proBNP, N-terminal prohormone of B-type natriuretic peptide; NYHA, New York Heart Association; OACs, oral anticoagulants; SAM, systolic anterior motion; SCD, sudden cardiac death

**Table 2:** Procedure characteristics and in-hospital outcomes

Procedure	
Number of injected septal arteries	2.5 ± 1.3
Volume of ethanol, mL	4.1 ± 2.0
Peak CK, IU/L	1543.5 ± 436.3
In-hospital outcomes	
Death, n (%)	0 (0.0)
VT/VF, n (%)	0 (0.0)
Complete AV block, n (%)	2 (18.2)
New CIED implantation, n (%)	0 (0.0)
Stroke, n (%)	0 (0.0)
Cardiac tamponade, n (%)	0 (0.0)
New atrial fibrillation, n (%)	0 (0.0)

Data are expressed as mean ± standard deviation or number (%)

AV, atrioventricular; CIED, cardiac implantable electrical device; CK, creatine kinase; VF, ventricular fibrillation; VT, ventricular tachycardia

### Parameters of cine MRI before and after ASA

As summarized in Table 3, Max-IVSTd and Max-IVSTs were significantly reduced in a week after ASA compared to before (Max-IVSTd: 19.3 ± 4.7 mm before ASA vs. 18.3 ± 4.6 mm after ASA, *p* = 0.001; Max-IVSTs: 22.0 ± 4.7 mm before ASA vs. 20.9 ± 4.5 mm after ASA, *p* = 0.003). LVEF was significantly reduced at a week after ASA compared to before (72.5 ± 9.4 % before ASA vs. 65.2 ± 10.7 % after ASA, *p* = 0.033), whereas LVEDVI, LVESVI, LVMI, RVEDVI, RVESVI, and RVEF did not show significant change. A larger LGE mass (*p* = 0.001) and %LGE (*p* < 0.001) were observed after ASA.

**Table 3:** Cine and LGE MRI and MRA before and 1 week after the ASA

Cine MRI	Before ASA	After ASA	*p*
Max-IVSTd, mm	19.3 ± 4.3	18.3 ± 4.6	0.001
Max-IVSTs, mm	22.0 ± 4.7	20.9 ± 4.5	0.003
LVEDVI, mL/m^2^	76.3 ± 9.3	82.3 ± 25.1	0.337
LVESVI, mL/m^2^	21.0 ± 6.9	29.3 ± 15.8	0.083
LVEF, %	72.5 ± 9.4	65.2 ± 10.7	0.033
LVMI, g/m^2^	95.3 ± 27.6	97.5 ± 30.2	0.65
RVEDVI, mL/m^2^	48.1 ± 8.2	53.3 ± 12.1	0.26
RVESVI, mL/m^2^	15.7 ± 8.0	18.0 ± 6.3	0.34
RVEF, %	68.1 ± 12.4	65.9 ± 11.1	0.55
LGE MRI			
LGE mass, g	17.4 ± 17.7	38.3 ± 26.7	0.001
%LGE, %	10.3 ± 6.0	22.6 ± 8.4	< 0.001
3D MRA			
AAO-D, mm	36.9 ± 7.5	36.6 ± 7.3	0.22
AAO-DI, mm/m^2^	23.9 ± 6.0	23.9 ± 5.9	0.82

Data are expressed as mean ± standard deviation

%LGE, a fraction of late gadolinium enhancement to left ventricle mass; AAO-D, ascending aortic-diameter; AAO-DI, ascending aortic-diameter index; ASA, alcohol septal ablation; LGE, late gadolinium enhancement; LVEDVI, left ventricular end-diastolic volume index; LVEF, left ventricular ejection fraction; LVESVI, left ventricular end-systolic volume index; LVMI, left ventricular mass index; Max-IVSTd, maximum interventricular septal thickness in diastole; Max-IVSTs, maximum interventricular septal thickness in systole; MRI, magnetic resonance imaging; MRA, magnetic resonance angiography, RVEDVI, right ventricular end-diastolic volume index; RVEF, right ventricular ejection fraction; RVESVI, right ventricular end-systolic volume index

### Ascending aortic flow and intra‐left ventricular flow patterns before and after ASA

Figures 2 and 4 show a representative case of changes in AAO and intra-LV flow patterns before and after ASA (Additional file 2–7: Movies 1–6). The characteristics of blood flow in the AAO and the LV in each patient are presented in Tables 4 and 5. As summarized in Table 6, vortex and helix flow patterns in AAO were less observed after ASA (1.45 ± 0.52 before ASA vs. 1.09 ± 0.30 after ASA, *p* = 0.046); however, the incidence of sole vortex or helix flow patterns did not significantly decrease after ASA. The duration as well as phase count of vortex and helix flow patterns in AAO shortened after ASA (duration: 744 ± 291 ms before ASA vs. 467 ± 258 ms after ASA, *p* < 0.001; phase count: 14.8 ± 4.4 phases before ASA vs. 10.5 ± 5.8 phases after ASA, *p* < 0.001). Regarding the comparison of intra-LV flow patterns before and after ASA, the diastolic anterior vortex area after ASA was larger than that before ASA (1628 ± 420 mm^2^ before ASA vs. 2974 ± 539 mm^2^ after ASA, *p* = 0.009); however, there were no significant changes in the diastolic posterior vortex area after ASA. The incidence of systolic vortex flow just above the LVOT showed a significant decrease after ASA compared to that before ASA (63.6% before ASA vs. 9.1% after ASA, *p* = 0.014). There were no changes in WSS_outer_ (*p* = 0.575) and WSS_inner_ (*p* = 0.949) between before and after ASA.

**Table 4:** Characteristics of blood flow in the ascending aorta before and after ASA

Case	Vortex		Helix		V + H		Vortex area, mm^2^		Vortex/Helix duration, ms		Vortex/Helix phase count, phases	
	Before ASA	After ASA	Before ASA	After ASA	Before ASA	After ASA	Before ASA	After ASA	Before ASA	After ASA	Before ASA	After ASA
1	1	1	1	1	2	2	5398	5477	818	405	18	9
2	1	1	0	0	1	1	8716	9464	1118	842	19	16
3	1	1	0	0	1	1	3724	3193	509	170	9	3
4	1	0	1	1	2	1	1804	0	1213	762	19	15
5	1	0	0	1	1	1	7896	0	608	515	15	12
6	1	1	1	0	2	1	5989	6062	657	600	15	12
7	1	0	1	1	2	1	9691	7037	706	507	17	15
8	1	1	0	0	1	1	16,244	15,230	1097	761	20	20
9	1	1	0	0	1	1	3742	5218	463	162	9	5
10	1	1	0	0	1	1	7145	6836	304	140	8	3
11	1	1	1	0	2	1	10,139	7488	688	278	14	5

ASA, alcohol septal ablation; V + H, vortex + helix

**Table 5:** Characteristics of blood flow in the left ventricle before and after ASA

Case	Diastole				Systole	
	Anterior vortex size, mm^2^		Posterior vortex size, mm^2^		Prevalence of vortex above the LVOT	
	Before ASA	After ASA	Before ASA	After ASA	Before ASA	After ASA
1	0	2920	0	2076	1	0
2	2679	3628	886	2750	1	1
3	0	0	3825	4146	0	0
4	3451	5463	458	380	1	0
5	2280	3270	333	616	1	0
6	0	0	0	0	1	0
7	1293	1424	0	0	1	0
8	1181	4166	1091	0	0	0
9	3894	3820	978	0	1	0
10	935	4679	0	354	0	0
11	2104	3345	592	610	0	0

ASA, alcohol septal ablation; LVOT, left ventricular outflow tract

**Table 6:** Comparison of hemodynamic parameters in the ascending aorta and intra-LV

	Before ASA n = 11	After ASA n = 11	*P*
AAO			
Vortex	11 (100%)	8 (73%)	0.083
Helix	5 (45%)	4 (36%)	0.564
Vortex + Helix	1.45 ± 0.52	1.09 ± 0.30	0.046
Vortex area, mm^2^	7317 ± 3964	6000 ± 4265	0.117
Vortex/Helix duration, ms	744 ± 291	467 ± 258	< 0.001
Vortex/Helix phase count, phases	14.8 ± 4.4	10.5 ± 5.8	< 0.001
WSS_outer_, Pa	1.15 ± 0.37	1.07 ± 0.56	0.575
WSS_inner_, Pa	0.88 ± 0.21	0.87 ± 0.37	0.949
LV			
Diastolic anterior vortex area, mm^2^	1628 ± 420	2974 ± 539	0.009
Diastolic posterior vortex area, mm^2^	458 (0–978)	380 (0–2076)	0.374
Systolic LVOT vortex	7 (63.6%)	1 (9.1%)	0.014

Data are expressed as mean ± standard deviation or median (interquartile range)

ASA, alcohol septal ablation; AAO, ascending aorta; LV, left ventricle; LVOT, left ventricular outflow tract; WSS_inner_, wall shear stress at inner curvature of ascending aorta; WSS_outer_, wall shear stress at outer curvature of ascending aorta; CIED, cardiac implantable electrical device; CMR, cardiac magnetic resonance; HOCM, hypertrophic obstructive cardiomyopathy; ICD, implantable cardioverter defibrillator; LVOTO: left ventricular outflow tract obstruction; MRI, magnetic resonance imaging; MVO, mid-ventricular obstruction; WSS, wall shear stress; WSS_inner_, wall shear stress at inner curvature of aortic arch; WSS_outer_, wall shear stress at outer curvature of aortic arch; PPG, peak pressure gradient

### Correlation between the difference in hemodynamic parameters and severity of HOCM before and after ASA

**Fig. 5:**
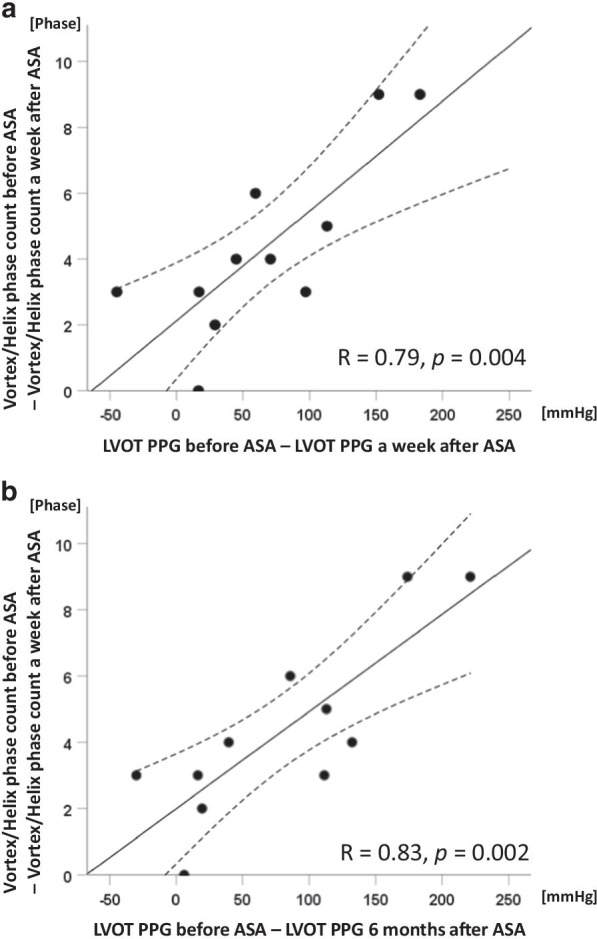
Correlation between the difference in LVOT PPG and the difference in vortex/helix phase count. The correlations are exhibited (**a**) between LVOT PPG before ASA—LVOT PPG a week after ASA and vortex/helix phase count before ASA—vortex/helix phase count a week after ASA and (**b**) between LVOT PPG before ASA—LVOT PPG 6 months after ASA and vortex/helix phase count before ASA—vortex/helix phase count a week after ASA. The solid and broken lines indicate the linear regression line and its confidence limits, respectively. Abbreviations: ASA, alcohol septal ablation; LVOT, left ventricular outflow tract; PPG, peak pressure gradient

As shown in Fig. 5a, the difference in LVOT PPG before and a week after ASA was significantly correlated with the difference in the phase count of vortex and helix flow in AAO before and a week after ASA (R = 0.79, *p* = 0.004). The difference in LVOT PPG before and 6 M after ASA was also significantly correlated with the difference in the phase count of vortex and helix flow in AAO before and a week after ASA (Fig. 5b, R = 0.83, *p* = 0.002).

### Interobserver variability

Interobserver variability of flow pattern in AAO (kappa = 0.75), duration of Vortex/Helix (ICC = 0.88), diastolic LV vortex area (ICC = 0.71), and the prevalence of systolic vortex (kappa = 0.73) were excellent.

## Discussion

Our study focused on the differences in AAO and intra-LV flow evaluated by 4D flow MRI in drug-refractory HOCM before and after ASA. We demonstrated that ASA affected AAO flow and intra-LV flow patterns, and these changes were associated with the improvement of hemodynamics and alleviation of the symptoms.

4D flow MRI can visualize changes in hemodynamics of AAO and intra-LV, and previous studies have demonstrated a disease-specific flow pattern of AAO in valvular heart disease and cardiomyopathy [9, 11, 17]. However, there are few studies showing changes in hemodynamics of AAO and intra-LV after open-heart surgery or catheter intervention. ASA is an invasive treatment for drug-refractory HOCM to relieve heart failure symptoms and improve hemodynamics [18]. The improvement in NYHA class of heart failure, serum NT-proBNP levels, and SAM grade of mitral valve after ASA suggests the successful outcome of ASA in our study. TTE has been frequently used to evaluate the effect of ASA in patients with HOCM [15, 19–22]. TTE can show changes in LVOT PPG, degree of SAM, and septal thickness, but cannot show changes in AAO flow and intra-LV flow pattern. In the present study, the decreased incidence of the vortex and/or helix flow and the shorter duration of vortex or helix flow in the AAO, and a larger area in major vortex in the LV were observed. The decreased phase count of vortex or helix flow in AAO was associated with decreased LVOT PPG and helped in understanding the effect of ASA on systemic hemodynamics.

A considerable number of studies have assessed the hemodynamics of AAO in a bicuspid aortic valve (BAV) cohort using 4D flow MRI. The previously reported evidence included the derangement of flow pattern with the developed helical flow or eccentric flow, an increase of WSS, and abnormal tissue architecture in the dilated aortic wall according to increased WSS. These results suggest that 4D flow MRI-derived hemodynamic characteristics may be an additional diagnostic tool as an imaging biomarker for aortic valve disease and concomitant aortic dilation.

A previous study by Allen et al. [13] showed that there was higher grade helical flow in patients with HOCM compared with patients with non-obstructive HCM, and there was a significant correlation between helix grade and LVOT PPG. SAM of the mitral valve was associated with increased helix grade. These findings indicate that abnormal flow patterns in AAO can be observed in HOCM, which may be associated with LVOT PPG. However, in contrast to BAV, aortic dilation in HCM was rarely observed and had no relation with the extent of hypertrophy or LVOTO [23]. Another study also exhibited no significant difference in age-, sex-, and body surface area-adjusted norms of aortic size between patients with and without obstructive physiology in HCM [24]. Thus, the pathophysiological significance of the helical flow characteristics in terms of the impact on the non-dilated AAO wall in patients with HCM is uncertain [13]. Overall, abnormal flow patterns in AAO may be associated with the severity of HOCM, and any interventions to decrease LVOT PPG can also correct the flow pattern in AAO independent of aortopathy. However, the association has been published only in a single case report [25]. Although merely a speculation, the mechanism of AAO vortex or helix flow in HOCM appears to be the shift of outflow jet apart from the centerline of aorta producing the helix or vortex flow. As supporting evidence for this speculation, the significant association between the severity of aortic stenosis and severity of vortex and helix flow [10] as well as the mitigation of vortex and helix flow in BAV after aortic valve replacement [26] have been reported.

Other factors associated with aberrant flow in AAO include aortic stenosis and regurgitation [11] and aortic aneurysm [27]. Considering these previous reports, no subject in our study revealed aortic valve disease. Although the cause-effect relation with HCM is uncertain, four patients in our study had mildly dilated AAO with a diameter of > 40 mm. Interestingly, the duration of vortex or helix flow was mostly reduced after ASA even in these four patients (Table 4: cases 2, 7, 8, and 11). We expected a significant reduction in WSS after ASA with the decrease in LVOT PPG; however, we did not observe a significant change. Although it is only a speculation, the healthy aortic valve might have cleared the aberrant flow of HOCM in the AAO to some extent, inhibited the WSS elevation, and avoided the AAO dilatation.

Intra-LV vortex flow has been reported in some studies [17, 28, 29]. In general, the vortex ring is generated at the mitral valve tip in diastole with LV inflow [29]. In our study, the anterior side of the vortex ring grew and expanded into the LV as a major vortex in diastole (Fig. 4a and b). The large vortex in the dilated LV has been reported in some previous studies [17, 28]. However, to the best of our knowledge, there is no previous report concerning the diastolic LV vortex in HOCM. Although the prevalence of diastolic intra-LV vortex was 100 % in patients with preserved and impaired LV function in a previous study [17], it was 72.7 % in the current study (Table 5). Compared to the study by Maron et al. [30], mitral valve was equally elongated in our cohort. Considering that the diastolic anterior vortex was generated at the tip of the mitral valve by flow separation, an elongated mitral valve leaflet in contact with the ventricular septum might attenuate the flow separation, resulting in a lower prevalence of diastolic intra-LV vortex. The other explanation is that the smaller LV cavity in HOCM might disturb the prevalence of the intra-LV vortex. Furthermore, we found a significant enlargement of the anterior vortex after ASA. The altered structure and chamber space in the LV cavity may restore the diastolic function, and reduced intra-LV and intra-LA pressure might change the LV inflow and subsequent vortex formation. Because the diastolic intra-LV vortex was considered efficient for preserving the hemodynamic energy [31], ASA might help in ameliorating the diastolic hemodynamic function in addition to improving the LVOTO. Although systolic rotational flow circulating the entire cavity of LV has been described in patients with impaired LV function [32], systolic vortex flow above LVOT has not been reported to date. Considering the significant decrease after ASA, systolic vortex flow just above LVOT and beneath aortic valve is probably associated with LVOTO and is a potential indicator for a favorable therapeutic effect.

The study limitations include the small sample size in the entire cohort and the lack of a control subject. Because the maximum flow velocity in the LVOT was elevated, 4D flow MRI was performed with relatively high VENC, which might have missed the blood flow with low velocity. The relatively low temporal resolution of 48 ± 6 ms could have missed the peak systole, resulting in an underestimation of peak flow velocity and WSS.

## Conclusions

In conclusion, less vortex or helix flow in the AAO and larger diastolic vortex flow in the LV were observed after ASA using 4D flow MRI. The elucidation of hemodynamics in HOCM before and after ASA helps us to understand the association between pathophysiology and manifestation of the disease and suggests a potential for outcome prediction, resulting in improved patients’ management. Further investigation with a longitudinal study design and a larger sample size is warranted to verify the association between the improvement of aberrant hemodynamics and outcome after ASA in HOCM.

## Supplementary information

**Additional file 1: Table S1:** Raw data for baseline characteristics, echocardiography, and cardiac MRI. The raw data from each patient are listed.

**Additional file 2: Movie 1:** Streamline movie of ascending aorta before ASA. In the ascending aorta, vortex flow in systole is followed by helical flow, which continues up to late diastole.

**Additional file 3: Movie 2:** Streamline movie of the ascending aorta a week after ASA. Although similar vortex flow in systole is observed, helical flow disappeared in early diastole at a week after ASA.

**Additional file 4: Movie 3:** Streamline movie of LV before ASA. Before ASA, the streamlines exhibit relatively small vortex in LV at late diastole.

**Additional file 5: Movie 4:** Streamline movie of LV a week after ASA. The late diastolic vortex in LV became larger at a week after ASA compared to that before ASA.

**Additional file 6: Movie 5:** Streamline movie of LVOT before ASA. Before ASA, the streamlines exhibit vortex flow above LVOT at peak systole.

**Additional file 7: Movie 6:** Streamline movie of LVOT after ASA. After ASA, the peak systolic vortex flow above LVOT disappeared.

## Data Availability

The datasets supporting the conclusions of this article are included within the article and its additional files.

## Abbreviations

%LGE: Fraction of late gadolinium enhancement to LV mass
2D: Two-dimensional
4D: Four-dimensional
6 M: Six months
AAO: Ascending aorta
ASA: Alcohol septal ablation
BAV: Bicuspid aortic valve
CIED: Cardiac implantable electrical device
FA: Flip angle
FIESTA: Fast imaging employing steady-state acquisition
FOV: Field of view
FSPGR: Fast spoiled gradient recalled acquisition in the steady state
HCM: Hypertrophic cardiomyopathy
HOCM: Hypertrophic obstructive cardiomyopathy
ICC: Intraclass correlation coefficients
LAD: Left atrial dimension
LAVI: Left atrial volume index
LGE: Late gadolinium enhancement
LV: Left ventricle
LVEDD: Left ventricular end-diastolic dimension
LVEDVI: Left ventricular end-diastolic volume index
LVEF: Left ventricular ejection fraction
LVESD: Left ventricular end-systolic dimension
LVESVI: Left ventricular end-systolic volume index
LVMI: Left ventricular mass index
LVOT: Left ventricular outflow tract
LVOTO: Left ventricular outflow tract obstruction
LVSVI: Left ventricular stroke volume index
Max-IVST: Maximum interventricular septal thickness
Max-IVSTd: Maximum interventricular septal thickness in diastole
Max-IVSTs: Maximum interventricular septal thickness in systole
MR: Mitral regurgitation
MRA: Magnetic resonance angiography
MRI: Magnetic resonance imaging
NEX: Number of excitations
NT-proBNP: N-terminal prohormone of B-type natriuretic peptide
NYHA: New York Heart Association
PPG: Peak pressure gradient
RBW: Receiver bandwidth
RV: Right ventricle
RVEDVI: Right ventricular end-diastolic volume index
RVEF: Right ventricular ejection fraction
RVESVI: Right ventricular end-systolic volume index
SAM: Systolic anterior motion
SD: Standard deviation
TE: Echo time
TR: Repetition time
TTE: Transthoracic echocardiography
VENC: Velocity sensitivity
WSS: Wall shear stress
WSS_inner_: Wall shear stress at inner curvature of aortic arch
WSS_outer_: Wall shear stress at outer curvature of aortic arch
