# Supplementary material for: Hemodynamic change in patients with hypertrophic obstructive cardiomyopathy before and after alcohol septal ablation using 4D flow magnetic resonance imaging: a retrospective observational study
Source: BMC Cardiovasc Disord. 2021 Apr 20;21:198. doi: 10.1186/s12872-021-02003-8 (PMC8059221; doi:10.1186/s12872-021-02003-8)
Supplement: Supplementary file 1 — Additional file 1: Table S1: Raw data for baseline characteristics, echocardiography, and cardiac MRI. The raw data from each patient are listed. [file 12872_2021_2003_MOESM1_ESM.docx]

| **Additional Table 1. Raw data for baseline characteristics, echocardiography, and cardiac MRI** | | | | | | | | | | | | | | | |  | |
| --- | --- | --- | --- | --- | --- | --- | --- | --- | --- | --- | --- | --- | --- | --- | --- | --- | --- |
| **Case number** | Age, years | Male | BMI, kg/m^2^ | NYHA III/IV | Syncope | HTN | DM | LD | COPD | Current smoking | CPA | Stroke | PAF | CIED | FH of SCD | |  |
| 1 | 78 | 0 | 24.0 | 1 | 0 | 1 | 0 | 0 | 0 | 0 | 0 | 0 | 0 | 0 | 0 | |  |
| 2 | 79 | 0 | 20.0 | 1 | 0 | 0 | 0 | 0 | 0 | 0 | 0 | 0 | 0 | 0 | 0 | |  |
| 3 | 45 | 1 | 25.7 | 1 | 0 | 0 | 0 | 0 | 0 | 0 | 0 | 0 | 1 | 0 | 0 | |  |
| 4 | 54 | 0 | 24.8 | 1 | 0 | 0 | 0 | 1 | 0 | 0 | 0 | 0 | 0 | 0 | 0 | |  |
| 5 | 77 | 0 | 20.9 | 1 | 1 | 0 | 0 | 0 | 0 | 0 | 0 | 0 | 0 | 0 | 0 | |  |
| 6 | 63 | 1 | 26.4 | 1 | 0 | 0 | 0 | 1 | 0 | 0 | 0 | 0 | 0 | 0 | 0 | |  |
| 7 | 70 | 0 | 20.4 | 0 | 0 | 1 | 0 | 1 | 0 | 0 | 0 | 0 | 1 | 0 | 0 | |  |
| 8 | 81 | 1 | 19.0 | 0 | 0 | 1 | 0 | 1 | 0 | 0 | 0 | 0 | 0 | 0 | 0 | |  |
| 9 | 56 | 0 | 23.0 | 1 | 0 | 0 | 0 | 0 | 0 | 0 | 0 | 0 | 0 | 0 | 1 | |  |
| 10 | 61 | 0 | 20.2 | 1 | 0 | 1 | 0 | 0 | 0 | 0 | 0 | 0 | 0 | 0 | 0 | |  |
| 11 | 75 | 0 | 22.4 | 0 | 0 | 0 | 0 | 0 | 0 | 0 | 0 | 0 | 0 | 0 | 0 | |  |

|  |  |  |  |  |  |  | |  | |  | |  | |  | |  | |  | |  | |  |
| --- | --- | --- | --- | --- | --- | --- | --- | --- | --- | --- | --- | --- | --- | --- | --- | --- | --- | --- | --- | --- | --- | --- |
| **Case**  **number** | FH  of HCM | Na  channel blockers | Beta blockers | Calcium channel blockers | ACE-Is / ARBs | | Amiodarone | | OACs | | NT-proBNP, pg/mL | | **TTE　before ASA** | | LVEDD, mm | | LVESD, mm | | Max-IVST, mm | | LAD, mm | |
| 1 | 0 | 1 | 1 | 1 | 0 | | 0 | | 0 | | 1541.0 | |  | | 41.6 | | 20.5 | | 19.1 | | 40.4 | |
| 2 | 0 | 0 | 1 | 0 | 0 | | 0 | | 0 | | 1114.0 | |  | | 42.8 | | 27.1 | | 15.1 | | 33.8 | |
| 3 | 0 | 0 | 1 | 0 | 0 | | 1 | | 1 | | 999.0 | |  | | 49.5 | | 23.1 | | 16.9 | | 52.4 | |
| 4 | 1 | 1 | 1 | 1 | 0 | | 0 | | 0 | | 211.0 | |  | | 41.8 | | 24.6 | | 13.5 | | 38.8 | |
| 5 | 0 | 1 | 0 | 0 | 0 | | 0 | | 0 | | 1940.0 | |  | | 40.2 | | 23.9 | | 15.6 | | 38.8 | |
| 6 | 0 | 1 | 1 | 1 | 1 | | 0 | | 0 | | 858.0 | |  | | 41.5 | | 33.1 | | 29.4 | | 44.6 | |
| 7 | 0 | 1 | 1 | 1 | 0 | | 0 | | 1 | | 970.0 | |  | | 45.0 | | 28.8 | | 18.4 | | 36.7 | |
| 8 | 0 | 1 | 1 | 1 | 0 | | 0 | | 0 | | 919.0 | |  | | 42.3 | | 24.8 | | 14.9 | | 36.6 | |
| 9 | 1 | 1 | 1 | 1 | 0 | | 0 | | 0 | | 5060.0 | |  | | 39.2 | | 22.9 | | 15.5 | | 41.7 | |
| 10 | 1 | 0 | 1 | 1 | 0 | | 0 | | 0 | | 1277.0 | |  | | 33.3 | | 21.0 | | 15.1 | | 37.2 | |
| 11 | 0 | 1 | 1 | 0 | 0 | | 0 | | 0 | | 2804.0 | |  | | 38.5 | | 21.0 | | 20.1 | | 44.0 | |

|  |  | |  | |  | |  | |  | |  | |  | |  | |  |  | |  | |  | |  |
| --- | --- | --- | --- | --- | --- | --- | --- | --- | --- | --- | --- | --- | --- | --- | --- | --- | --- | --- | --- | --- | --- | --- | --- | --- |
| **Case**  **number** | LAVI, mL/m^2^ | Resting LVOT PPG, mmHg | | Moderate or  Severe MR | | Ant. leaflet length of MV, mm | | Post. leaflet length of MV, mm | | SAM of MV | | **TTE　1W after ASA** | | LVEDD, mm | | LVESD, mm | | | Max-IVST, mm | | LAD, mm | | LAVI, mL/m^2^ | |
| 1 | 69.5 | 236.0 | | 0 | | 21.8 | | 18.2 | | G3 | |  | | 41.8 | | 27.9 | | | 17.8 | | 51.2 | | 60.1 | |
| 2 | 43.1 | 129.0 | | 1 | | 23.5 | | 16.4 | | G3 | |  | | 47.8 | | 28 | | | 14.7 | | 39.7 | | 49.4 | |
| 3 | 89.2 | 97.5 | | 1 | | 31.9 | | 17.4 | | G4 | |  | | 44.1 | | 20 | | | 15.6 | | 39.5 | | 68.9 | |
| 4 | 34.0 | 100.0 | | 1 | | 24.3 | | 14.1 | | G0 | |  | | 47.4 | | 28.5 | | | 12.2 | | 38.4 | | 44 | |
| 5 | 49.0 | 33.0 | | 0 | | 25.8 | | 19.0 | | G4 | |  | | 40.6 | | 24.6 | | | 15.6 | | 41.8 | | 52.9 | |
| 6 | 45.3 | 35.0 | | 0 | | 30.0 | | 19.7 | | G3 | |  | | 42.1 | | 33.3 | | | 29 | | 43.6 | | 34.8 | |
| 7 | 71.6 | 38.0 | | 1 | | 26.6 | | 25.6 | | G3 | |  | | 45.7 | | 26 | | | 17.8 | | 45.7 | | 49.5 | |
| 8 | 46.9 | 41.0 | | 1 | | 25.0 | | 21.6 | | G2 | |  | | 48.3 | | 31.8 | | | 15 | | 35.6 | | 27.8 | |
| 9 | 63.8 | 157.2 | | 0 | | 34.0 | | 14.3 | | G4 | |  | | 42.6 | | 24.2 | | | 15.7 | | 39.8 | | 53.5 | |
| 10 | 57.3 | 140.0 | | 1 | | 17.1 | | 11.4 | | G3 | |  | | 36.1 | | 24.7 | | | 15.1 | | 34.7 | | 52.9 | |
| 11 | 77.6 | 189.0 | | 1 | | 25.0 | | 14.1 | | G4 | |  | | 34.3 | | 21.8 | | | 20.8 | | 44.2 | | 90.6 | |

|  |  |  | |  | |  |  | |  | |  | |  | |  | |  | |  | |  | |  |
| --- | --- | --- | --- | --- | --- | --- | --- | --- | --- | --- | --- | --- | --- | --- | --- | --- | --- | --- | --- | --- | --- | --- | --- |
| **Case**  **number** | Resting LVOT PPG, mmHg | | Moderate or  Severe MR | | SAM of MV | **TTE　6M after ASA** | | LVEDD, mm | | LVESD, mm | | Max-IVST, mm | | LAD, mm | | LAVI, mL/m^2^ | | Resting LVOT PPG, mmHg | | Moderate or  Severe MR | | SAM of MV | |
| 1 | 53.0 | | 0 | | G3 |  | | 38.3 | | 24.3 | | 17.0 | | 46.0 | | 65.9 | | 14.7 | | 0 | | G2 | |
| 2 | 32.0 | | 0 | | G3 |  | | 30.3 | | 43.4 | | 14.1 | | 30.3 | | 56.2 | | 17.6 | | 0 | | G2 | |
| 3 | 38.0 | | 1 | | G3 |  | | 44.1 | | 26.6 | | 15.6 | | 45.4 | | 76.2 | | 11.6 | | 0 | | G2 | |
| 4 | 55.0 | | 0 | | G0 |  | | 36.8 | | 20.7 | | 12.2 | | 41.9 | | 28.2 | | 60.4 | | 0 | | G0 | |
| 5 | 77.9 | | 0 | | G4 |  | | 40.6 | | 21.6 | | 12.6 | | 39.0 | | 55.3 | | 63.0 | | 0 | | G4 | |
| 6 | 18.0 | | 0 | | G3 |  | | 50.8 | | 41.3 | | 24.8 | | 42.8 | | 34.9 | | 18.7 | | 0 | | G2 | |
| 7 | 9.0 | | 0 | | G2 |  | | 44.6 | | 30.3 | | 16.0 | | 33.3 | | 60.7 | | 18.5 | | 0 | | G2 | |
| 8 | 24.4 | | 0 | | G1 |  | | 44.8 | | 29.1 | | 14.0 | | 38.8 | | 36.9 | | 35.0 | | 0 | | G1 | |
| 9 | 86.5 | | 0 | | G3 |  | | 39.1 | | 24.5 | | 14.8 | | 39.6 | | 42.6 | | 25.0 | | 0 | | G2 | |
| 10 | 27.0 | | 0 | | G2 |  | | 43.0 | | 24.9 | | 9.6 | | 39.1 | | 45.4 | | 27.0 | | 0 | | G1 | |
| 11 | 37.0 | | 0 | | G3 |  | | 44.1 | | 29.7 | | 21.1 | | 40.7 | | 95.8 | | 15.0 | | 0 | | G2 | |

|  |  |  |  |  |  |  |  |  |  |  |  |
| --- | --- | --- | --- | --- | --- | --- | --- | --- | --- | --- | --- |
| **Case**  **number** | **Procedural** | Number of injected septal arteries | Volume of ethanol, mL | Peak CK, IU/L | **In-hospital Outcomes** | Death | VT/VF | Complete AV block | New CIED Implantation | Stroke | Cardiac tamponade |
| 1 |  | 1 | 1.8 | 976 |  | 0 | 0 | 0 | 0 | 0 | 0 |
| 2 |  | 5 | 6.2 | 1427 |  | 0 | 0 | 0 | 0 | 0 | 0 |
| 3 |  | 2 | 3.5 | 1073 |  | 0 | 0 | 0 | 0 | 0 | 0 |
| 4 |  | 1 | 1.5 | 2043 |  | 0 | 0 | 0 | 0 | 0 | 0 |
| 5 |  | 2 | 3.8 | 1852 |  | 0 | 0 | 1 | 0 | 0 | 0 |
| 6 |  | 3 | 5.5 | 2078 |  | 0 | 0 | 0 | 0 | 0 | 0 |
| 7 |  | 3 | 3.5 | 1227 |  | 0 | 0 | 1 | 0 | 0 | 0 |
| 8 |  | 1 | 1.6 | 1103 |  | 0 | 0 | 0 | 0 | 0 | 0 |
| 9 |  | 4 | 7.5 | 1346 |  | 0 | 0 | 0 | 0 | 0 | 0 |
| 10 |  | 3 | 4.5 | 1716 |  | 0 | 0 | 0 | 0 | 0 | 0 |
| 11 |  | 3 | 5.4 | 2138 |  | 0 | 0 | 0 | 0 | 0 | 0 |

|  |  |  | |  |  | |  | |  | |  | |  | |  | |  | |  | |  | |  | |  | |  |
| --- | --- | --- | --- | --- | --- | --- | --- | --- | --- | --- | --- | --- | --- | --- | --- | --- | --- | --- | --- | --- | --- | --- | --- | --- | --- | --- | --- |
| **Case**  **number** | New AF | **Cine MRI Before ASA** | Max-IVSTd, mm | | | Max-IVSTs, mm | | LVEDVI, mL/m^2^ | | LVESVI, mL/m^2^ | | LVEF, % | | LVMI, g/m^2^ | | RVEDVI, mL/m^2^ | | RVESVI, mL/m^2^ | | RVEF, % | | **LGE MRI Before ASA** | | LGE mass, g | | %LGE, % | |
| 1 | 0 |  | 21.5 | | | 26.7 | | 71.9 | | 12.4 | | 83.0 | | 86.6 | | 42.2 | | 9.8 | | 77.0 | |  | | 24.5 | | 17.6 | |
| 2 | 0 |  | 18.1 | | | 21.7 | | 79.8 | | 22.4 | | 72.0 | | 99.4 | | 54.1 | | 18.5 | | 66.0 | |  | | 8.3 | | 6.9 | |
| 3 | 0 |  | 20.1 | | | 21.9 | | 87.5 | | 26.0 | | 70.0 | | 73.5 | | 53.6 | | 10.2 | | 81.0 | |  | | 6.2 | | 4.2 | |
| 4 | 0 |  | 12.7 | | | 15.1 | | 79.0 | | 15.5 | | 80.0 | | 55.0 | | 55.6 | | 21.1 | | 62.0 | |  | | 3.5 | | 4.2 | |
| 5 | 0 |  | 15.3 | | | 16.8 | | 70.1 | | 14.2 | | 80.0 | | 95.9 | | 38.4 | | 14.0 | | 64.0 | |  | | 5.2 | | 3.9 | |
| 6 | 0 |  | 29.1 | | | 31.6 | | 65.0 | | 18.4 | | 72.0 | | 163.4 | | 46.9 | | 22.1 | | 53.0 | |  | | 67.0 | | 22.8 | |
| 7 | 0 |  | 18.6 | | | 20.4 | | 91.3 | | 27.6 | | 70.0 | | 110.7 | | 41.3 | | 14.8 | | 64.0 | |  | | 20.2 | | 13.0 | |
| 8 | 0 |  | 16.1 | | | 19.1 | | 84.9 | | 18.6 | | 78.0 | | 84.3 | | 65.3 | | 34.1 | | 48.0 | |  | | 16.2 | | 13.0 | |
| 9 | 0 |  | 21.2 | | | 22.4 | | 68.8 | | 35.8 | | 48.0 | | 87.2 | | 46.2 | | 10.9 | | 76.0 | |  | | 14.6 | | 8.8 | |
| 10 | 0 |  | 17.7 | | | 19.8 | | 63.3 | | 16.4 | | 74.0 | | 110.3 | | 39.6 | | 13.2 | | 67.0 | |  | | 14.8 | | 11.2 | |
| 11 | 0 |  | 22.3 | | | 26.2 | | 77.5 | | 23.3 | | 70.0 | | 82.3 | | 46.2 | | 4.3 | | 91.0 | |  | | 10.5 | | 7.4 | |

|  |  |  | |  | |  | |  | | |  | |  | |  | |  | |  | |  | |  |  | |  |
| --- | --- | --- | --- | --- | --- | --- | --- | --- | --- | --- | --- | --- | --- | --- | --- | --- | --- | --- | --- | --- | --- | --- | --- | --- | --- | --- |
| **Case**  **number** | **3D MRA Before ASA** | | AAO-D, mm | | AAO-DI, mm/m^2^ | | **Cine MRI After ASA** | | Max-IVSTd, mm | Max-IVSTs, mm | | LVEDVI, mL/m^2^ | | LVESVI, mL/m^2^ | | LVEF, % | | LVM, g/m^2^ | | RVEDVI, mL/m^2^ | | RVESVI, mL/m^2^ | | | RVEF, % | |
| 1 |  | | 35.9 | | 23.9 | |  | | 20.5 | 25.6 | | 62.1 | | 14 | | 77.0 | | 91.3 | | 41.9 | | 9.8 | | | 77.0 | |
| 2 |  | | 42.6 | | 32.8 | |  | | 17.0 | 20.8 | | 79.3 | | 16.2 | | 80.0 | | 91.5 | | 49.9 | | 18.7 | | | 63.0 | |
| 3 |  | | 30.4 | | 15.9 | |  | | 18.2 | 21.3 | | 92.8 | | 25.4 | | 73.0 | | 66.4 | | 61.8 | | 30.4 | | | 51.0 | |
| 4 |  | | 29.2 | | 18.6 | |  | | 11.6 | 13.2 | | 67.5 | | 20.1 | | 70.0 | | 59.0 | | 61.1 | | 18.5 | | | 70.0 | |
| 5 |  | | 35.2 | | 26.4 | |  | | 13.1 | 15.9 | | 84.8 | | 19.5 | | 77.0 | | 96.8 | | 79.1 | | 22.1 | | | 72.0 | |
| 6 |  | | 32.1 | | 17.8 | |  | | 28.6 | 29.9 | | 62.9 | | 25.6 | | 59.0 | | 159.0 | | 40.6 | | 22.4 | | | 45.0 | |
| 7 |  | | 44.6 | | 28.9 | |  | | 17.4 | 18.0 | | 147 | | 71.4 | | 52.0 | | 146.0 | | 43.9 | | 15.7 | | | 64.0 | |
| 8 |  | | 53.0 | | 31.7 | |  | | 16.8 | 19.2 | | 81.4 | | 37.8 | | 54.0 | | 79.6 | | 56.0 | | 23.5 | | | 58.0 | |
| 9 |  | | 30.9 | | 17.7 | |  | | 20.1 | 22.3 | | 54.8 | | 27 | | 51.0 | | 99.8 | | 37.9 | | 11.9 | | | 69.0 | |
| 10 |  | | 31.6 | | 21.6 | |  | | 16.0 | 19.6 | | 75.7 | | 30.5 | | 60.0 | | 85.5 | | 56.3 | | 14.0 | | | 75.0 | |
| 11 |  | | 40.9 | | 27.8 | |  | | 21.7 | 23.4 | | 97 | | 35.3 | | 64.0 | | 97.7 | | 58.0 | | 11.3 | | | 81.0 | |

|  |  |  |  |  | |  | |  | |  | |  | |  | |  | |  | |  | |  |
| --- | --- | --- | --- | --- | --- | --- | --- | --- | --- | --- | --- | --- | --- | --- | --- | --- | --- | --- | --- | --- | --- | --- |
| **Case**  **number** | **LGE MRI After ASA** | LGE mass, g | %LGE, % | | **3D MRA After ASA** | | AAO-D, mm | | AAO-DI, mm/m^2^ | | **WSS Before ASA** | | WSS_outer_, Pa | | WSS_inner_, Pa | | **WSS After ASA** | | WSS_outer_, Pa | | WSS_inner_, Pa | |
| 1 |  | 28.2 | 20.3 | |  | | 35.7 | | 24.1 | |  | | 1.05 | | 0.49 | |  | | 1.26 | | 0.98 | |
| 2 |  | 17.0 | 14.6 | |  | | 43.0 | | 33.1 | |  | | 0.68 | | 0.67 | |  | | 0.78 | | 0.91 | |
| 3 |  | 27.1 | 18.5 | |  | | 29.8 | | 15.6 | |  | | 0.77 | | 1.03 | |  | | 0.67 | | 1.25 | |
| 4 |  | 10.0 | 12.1 | |  | | 27.9 | | 18.0 | |  | | 1.49 | | 0.77 | |  | | 2.53 | | 1.74 | |
| 5 |  | 19.4 | 14.3 | |  | | 34.4 | | 25.7 | |  | | 0.88 | | 1.13 | |  | | 1.20 | | 0.68 | |
| 6 |  | 106.0 | 37.2 | |  | | 32.9 | | 18.6 | |  | | 0.97 | | 0.85 | |  | | 0.60 | | 0.79 | |
| 7 |  | 49.0 | 29.2 | |  | | 42.8 | | 27.6 | |  | | 1.22 | | 0.83 | |  | | 0.69 | | 0.61 | |
| 8 |  | 34.7 | 24.5 | |  | | 52.4 | | 31.5 | |  | | 1.69 | | 1.10 | |  | | 1.29 | | 0.75 | |
| 9 |  | 44.8 | 25.3 | |  | | 30.5 | | 17.3 | |  | | 0.82 | | 1.14 | |  | | 0.57 | | 0.57 | |
| 10 |  | 27.0 | 17.7 | |  | | 32.9 | | 23.1 | |  | | 1.65 | | 0.94 | |  | | 1.21 | | 0.86 | |
| 11 |  | 58.1 | 35.0 | |  | | 40.2 | | 27.9 | |  | | 1.46 | | 0.68 | |  | | 0.97 | | 0.39 | |

Abbreviations: %LGE: a fraction of late gadolinium enhancement to left ventricle mass, AAO-D: ascending aortic-diameter, AAO-DI: ascending aortic-diameter index, ACE-Is: adenosine converting enzyme-inhibitors, AF: atrial fibrillation, ARBs: angiotensin Ⅱ receptor blockers, ASA: alcohol septal ablation; AV: atrioventricular, BMI: body mass index, CIED: cardiac implantable electrical device, CK: creatine kinase, COPD: chronic obstructive pulmonary disease, CPA: cardiopulmonary arrest, DM: diabetes mellitus, FH: family history, HCM, hypertrophic cardiomyopathy, HTN: hypertension, LAD: left atrial dimension, LAVI: left atrial volume index, LD: lipid disorder, LGE: late gadolinium enhancement, LVEDD: left ventricular end-diastolic dimension, LVEDVI: left ventricular end-diastolic volume index, LVEF: left ventricular ejection fraction, LVESD: left ventricular end-systolic dimension, LVESVI: left ventricular end-systolic volume index, LVMI: left ventricular mass index, LVOT PPG: peak pressure gradient at left ventricular outflow tract, Max-IVST: maximum interventricular septal thickness, Max-IVSTd: maximum interventricular septal thickness in diastole, Max-IVSTs: maximum interventricular septal thickness in systole, MR: mitral regurgitation, MRI: magnetic resonance imaging, MV: mitral valve, Na: natrium (sodium), NT-proBNP: N-terminal prohormone of brain B-type natriuretic peptide, NYHA: New York Heart Association, OACs: oral anticoagulants, PAF: paroxysmal atrial fibrillation, RVEDVI: right ventricular end-diastolic volume index, RVEF: right ventricular ejection fraction, RVESVI: right ventricular end-systolic volume index, SAM: systolic anterior motion, SCD: sudden cardiac death, TTE: transthoracic echocardiography, VF: ventricular fibrillation, VT: ventricular tachycardia, WSS: wall shear stress, WSS_inner_: wall shear stress at inner curvature of ascending aorta, WSS_outer_: wall shear stress at outer curvature of ascending aorta
